# It is not the parts, but how they interact that determines the behaviour of circadian rhythms across scales and organisms

**DOI:** 10.1098/rsfs.2013.0076

**Published:** 2014-06-06

**Authors:** Daniel DeWoskin, Weihua Geng, Adam R. Stinchcombe, Daniel B. Forger

**Affiliations:** 1 Department of Mathematics, University of Michigan, 2074 East Hall, 530 Church Street, Ann Arbor, MI 48109, USA; 2 Center for Computational Medicine and Bioinformatics, University of Michigan, 2074 East Hall, 530 Church Street, Ann Arbor, MI 48109, USA; 3 Department of Mathematics, Southern Methodist University, 135 Clements Hall, Dallas, TX 75275, USA

**Keywords:** systems biology, biological rhythms, circadian clocks, mathematical biology

## Abstract

Biological rhythms, generated by feedback loops containing interacting genes, proteins and/or cells, time physiological processes in many organisms. While many of the components of the systems that generate biological rhythms have been identified, much less is known about the details of their interactions. Using examples from the circadian (daily) clock in three organisms, *Neurospora*, *Drosophila* and mouse, we show, with mathematical models of varying complexity, how *interactions* among (i) promoter sites, (ii) proteins forming complexes, and (iii) cells can have a drastic effect on timekeeping. Inspired by the identification of many transcription factors, for example as involved in the *Neurospora* circadian clock, that can both activate and repress, we show how these multiple actions can cause complex oscillatory patterns in a transcription–translation feedback loop (TTFL). Inspired by the timekeeping complex formed by the NMO–PER–TIM–SGG complex that regulates the negative TTFL in the *Drosophila* circadian clock, we show how the mechanism of complex formation can determine the prevalence of oscillations in a TTFL. Finally, we note that most mathematical models of intracellular clocks model a single cell, but compare with experimental data from collections of cells. We find that refitting the most detailed model of the mammalian circadian clock, so that the coupling between cells matches experimental data, yields different dynamics and makes an interesting prediction that also matches experimental data: individual cells are bistable, and network coupling removes this bistability and causes the network to be more robust to external perturbations. Taken together, we propose that the interactions between components in biological timekeeping systems are carefully tuned towards proper function. We also show how timekeeping can be controlled by novel mechanisms at different levels of organization.

## 1. Introduction

Three levels of biochemical detail exist in the circadian timekeeping system of higher organisms, as well as many other rhythm generators: transcriptional regulation, post-translational modification and intercellular communication. For example, the mammalian daily (circadian) clock is controlled by the roughly 20 000 neurons of the suprachiasmatic nuclei (SCN), each of which has interlocked positive and negative genetic feedback loops in transcription and post-translational modifications that generate oscillations in the concentrations of core clock proteins with a period of 24 h. While many of the components of these timekeeping systems have been identified, how interactions between components at each of the three levels contribute to the generation of robust rhythms remains to be investigated. Here, we explore interactions at these three scales.

### 1.1. Transcriptional regulatory elements

Genetic negative feedback loops lie at the core of many rhythmic processes within cells, including those involved in circadian timekeeping [1,2], embryogenesis [3], cell cycle [4] and DNA damage repair [5]. For example, at the core of the mammalian circadian clock is a negative feedback loop in which a transcriptional activating complex (BMAL::CLOCK) binds to E-boxes, transcriptional regulatory sites, in the promoters of the *Per* and *Cry* genes, activating their transcription. The protein products of these genes then form complexes, which return to the nucleus to bind to and deactivate BMAL::CLOCK, thereby inhibiting their own production. While multiple E-box DNA-binding sites for BMAL::CLOCK exist [6], little is known about their interaction.

A similar feedback loop structure exists in the circadian clocks of *Drosophila* and *Neurospora* [7,8]. One interesting twist is that the core repressor of the *Neurospora* clock, FRQ, also can stabilize activators, causing it to act as an activator as well [9]. Another *Neurospora* transcription factor involved in circadian timekeeping, VIVID, also acts as both an activator and repressor [10]. The bacterial transcription factor araC, used in a synthetic clock [11], can act as either a repressor or an activator at different sites [12]. Many other transcription factors show this property [13]. Thus, another possibility could be that a protein could act as both an activator and repressor by binding to different sites, which is a motif that remains to be explored in models of circadian clocks.

### 1.2. Protein complex formation

Following transcriptional regulation, many important phosphorylation and complex formation steps occur in cellular rhythm-generating systems [14,15]. They are difficult to model, as the number of possible complexes grows exponentially in the number of protein species that can bind. For example, in a recent detailed model of the mammalian circadian clock by Kim & Forger [16], the core negative feedback loop of the model focused on only six different proteins. However, the model had to account for almost 150 different possible species once all complexes were included, as well as nuclear transport and the action of kinases.

One specific example of complex formation in rhythm generation can be found in the *Drosophila* circadian clock. The expressions of four proteins, PERIOD (PER), TIMELESS (TIM), NEMO (NMO) and SHAGGY (SGG), are all under the control of a transcriptional complex, formed by the CLK and CYC proteins, that binds rhythmically to genes with E-boxes [17]. PER, TIM, NMO and SGG form a complex, where post-translational modifications occur, which control the rhythmic activation by CLK and CYC [18,19]. A similar complex is created in the mammalian and *Neurospora* circadian clocks. An outstanding question is whether the mechanism of protein complex formation matters in rhythm generation.

### 1.3. Coupled cellular networks

Finally, in addition to intracellular dynamics, intercellular signalling between cells also plays an important role in cellular rhythm-generating systems [20]. For example, isolated neurons of the SCN show weak, noisy rhythms [21] that can gain or lose rhythmicity in response to external stimuli [22]. When coupled together, however, these neurons form a robust circuit with precise 24 h oscillations that is resistant to genomic mutations [23]. Thus, important interactions on the network level lead to emergent behaviours that cannot be seen by considering only a single cell.

Here, we extend the Kim & Forger model [16] of intracellular dynamics to make a detailed multicellular model of the SCN. We explore how a multicellular SCN model is able to reproduce differences in phenotype between dispersed SCN neurons and the SCN network, which shows robust oscillations less affected by genomic mutations as well as external stimuli.

### 1.4. Overall goals

While our simulations have implications for specific rhythm-generating systems, especially circadian timekeeping in higher organisms, our results show that many important details of the biochemical mechanisms underlying rhythm generation in many systems remain unexplored. This may be because these simulations often require advanced computing techniques, such as the use of graphics processing units (GPUs) employed here, to simulate detailed multicellular biochemical models. While GPU computing is not the only method available for performing these simulations, it is particularly well suited for simulating networks of biological cells as the models are easily parallelizable on GPU cards and require sharing information between cells, which can be done rapidly using the globally available memory on a GPU card. With models at three different scales, we find that the interaction mechanisms used in circadian clocks favour robust oscillations.

## 2. Interactions between promoter-binding sites

Genetic networks require fine-tuning and control, generally provided in the form of transcriptional regulation. Even in a simple eukaryote such as yeast, as many as 37% of promoters were found to bind transcriptional regulators, and of these, more than one-third bound two or more regulators [24]. While these regulatory sites are now easily identified using high throughput assays such as ChIPSeq [25], interactions between transcriptional regulators are much more difficult to characterize.

Here, we study transcriptional regulation in the context of a generalized feedback loop model based on that by Goodwin [26]. The model and corresponding equations are shown in box 1. We allow a general transcription rate, *f*(*P_L_*), which is a function of the concentration of a transcription factor *P_L_*. We model two sites where *P_L_* can bind. In the first model, there are two repressive sites that act independently, as seen, for example in the E-boxes in the *Per1* promoter [27]. It has previously been shown that complex behaviours, e.g. chaos, cannot be seen in such a simple system [28]. In the second model, two sites are again modelled, but one site acts to repress and one activates. Through this, we study how the interactions between transcriptional regulatory sites within a genetic feedback loop change oscillatory behaviour.

Box 1. **Description of a simple transcription–translation feedback loop model, with which we study the effects of transcriptional regulation mechanisms. A diagram of the feedback loop is shown, along with differential equations describing the dynamics of each of the protein species involved, and all parameters and initial conditions used.**

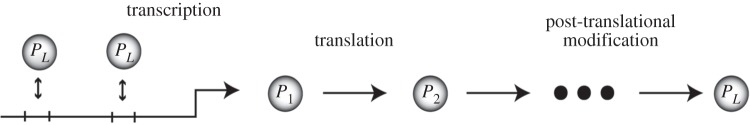

The model equations take the form

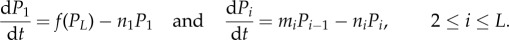

**Figure 1.**
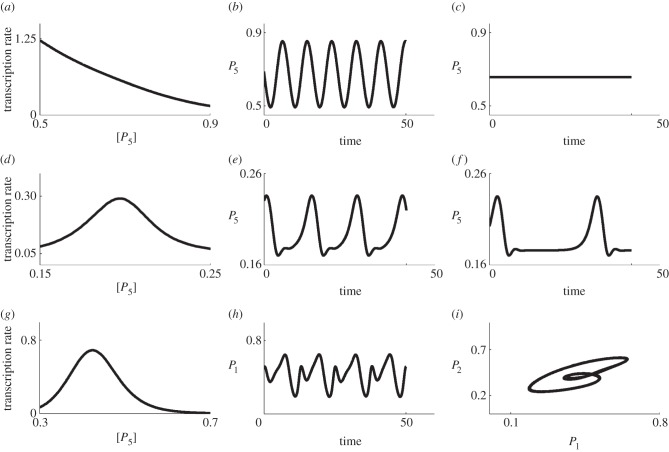
Changes in transcriptional regulation can drastically affect the behaviour of genetic oscillators. We consider three possible mechanisms of transcriptional regulation, where the rate of transcription as a function of a transcription factor is plotted in (*a*), (*d*) and (*g*) (box 1). Each assumes that there are two regions on a promoter where a transcription factor (*P_L_*) can bind. In the first model (shown in (*a*)), binding to either site can stop transcription. For this mechanism, the system can either show oscillations (*b*) or quiescence (*c*) depending on the initial state of the system. In models (*d*) and (*g*), binding at one site activates transcription and binding at the other site represses transcription. For model (*d*), small changes in the transcription regulation function can change the shape of oscillations and can lengthen the period (*e*–*f*). With a different choice of transcriptional regulation function, shown in (*g*), chaotic behaviour can also be seen (*h*–*i*). Rate constants and initial conditions can be found in box 1. For each simulation, an initial transient is not shown.

This simple model of a biochemical feedback loop tracks the concentration of transcripts from a gene (*P*_1_), and the concentration of protein translated from these transcripts. The protein can exist in several states (*P*_2_, …, *P_L_*) due to post-translational modifications that eventually allow the protein to act as a transcription factor (*P_L_*). The constant *n*_1_ is the degradation rate of the transcript, and for *i* > 1, *n_i_* is the sum of the rate of clearance for protein state *P_i_* and the conversion rate of protein state *P_i_* into the next state, *P_i_*_+1_. Similarly, *m*_2_ is the rate of translation of the protein, and *m_i_* (*i* > 2) is the rate of conversion of protein *P_i−_*_1_ into protein *P_i_*. Here, we choose *L* = 5 and all rate constants to be 1. We also assume that there are two binding sites for *P*_5_ on the gene. For simulations in figure 1*b*,*c*, the transcription rate (shown in figure 1*a*) was chosen to be

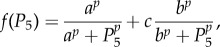
where *a* = 0.5, *b* = 0.75, *c* = 0.75 and *p* = 8. In figure 1*b*, all initial conditions were chosen to be 0.5, and in figure 1*c* all initial conditions were chosen to be 0.7. In figure 1*d*–*i*, the transcriptional regulation function was of the form

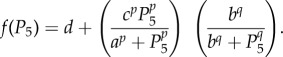

For figure 1*b*, *p* = 10, *q* = 25, *a* = 2, *b* = 0.2 and *c* = 9.25446. In figure 1*e*, *d* was chosen to be 0.055 and in figure 1*h* it was chosen to be 0.052826. These parameters were specifically chosen to show that a small change in parameters can yield a large change in behaviour. The initial conditions for figure 1*e*,*h* were that all variables started at values of 0.176. In figure 1*c*, *p* = 10, *q* = 13, *c* = 1.0824, *a* = 0.423, *b* = 0.44 and *d* was chosen to be 0. The initial conditions for figure 1*f*,*i* were *P*_1_ = 0.07, *P*_2_ = 0.4, *P*_3_ = 0.8, *P*_4_ = 0.2 and *P*_5_ = 0.5.

We searched for different types of oscillatory behaviour with different mechanisms of transcriptional regulation. To our surprise, we saw many types of behaviour, which had not yet been classified in genetic networks with just one feedback loop (although several had been seen in more complex networks [29,30]). One behaviour is called bistability, or hard excitation (figure 1*a*–*c*), where a stable steady state and a stable oscillatory state coexist [31]. The feedback loop displays either oscillations (figure 1*b*) or quiescence (figure 1*c*) depending on the initial conditions. In this model, oscillations can be started or stopped by external signals to the genetic network. This is a hallmark of many circadian clocks [29,32] and was found with the model of two repressive sites.

Allowing for interactions between an activator and repressor site greatly increases the behaviours that are possible. For example, during the course of an oscillation, the system comes very close to an equilibrium point (figure 1*f*), slowing as it approaches the equilibrium point, causing the oscillations to develop a long period. This is one example where small changes in transcription rate change the shape of the time course of oscillations by controlling how long the oscillator spends near an equilibrium point. This additional equilibrium point could not be seen in the model of two repressive sites, because the transcription regulation function was monotonic.

Finally, we note that more complex behaviours including chaos, with sensitive dependence on initial conditions, can also be seen (figure 1*g*–*i*) with an activator and repressor sites. This is not possible in the model of two repressive sites [28]. These results show that it is not sufficient to simply know that a given transcription factor binds to a regulatory site on a gene. On the contrary, the mechanism of interaction between transcriptional regulatory sites can greatly influence the rhythmic behaviours created in a genetic feedback loop.

## 3. Interactions between proteins in complex formation

We next consider the mechanisms of post-translational modification, in particular complex formation. Inspired by the *Drosophila* circadian clock (see Introduction), we begin by considering a general system of *N* distinct proteins (numbered 1 through *N*) that may combine to form complexes. Initially, the protein monomers are unbound, and we assume that proteins may not be repeated within a complex. We analyse the binding dynamics seen under three different models of complex formation:

— Model 1—individual proteins must bind one at a time to other individual proteins or existing complexes to create new complexes;
— Model 2—any individual protein or existing complex can bind to any other as long as no proteins are repeated within a complex; and
— Model 3—only proteins with consecutive indices can form a link in order to form a complex (i.e. 1 can only bind to 2, 2 can only bind to 1 or 3, 3 can only bind to 2 or 4, etc.).

**Figure 2.**
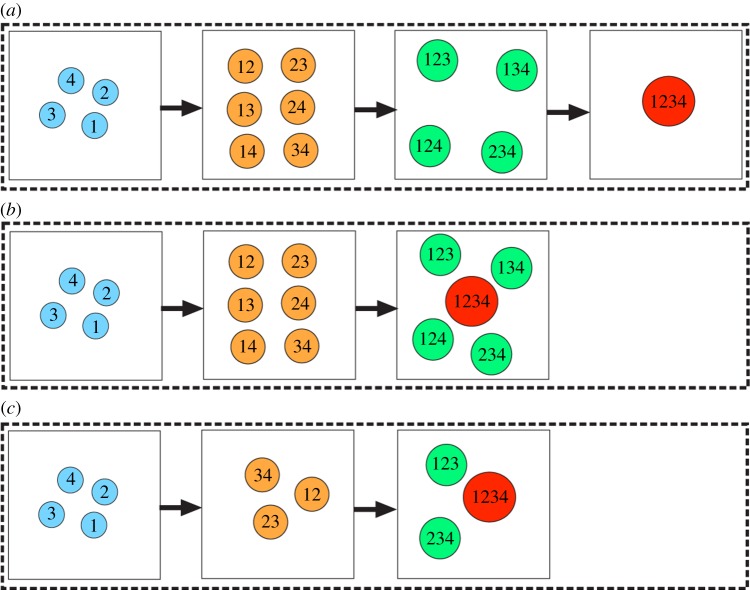
Three generic models of complex formation. Here, we consider four proteins, which can bind by several mechanisms of complex formation. In (*a*), we assume that any protein complex can bind only with individual proteins that are not part of the complex. In (*b*), we assume that any complex can bind with any other complex so long as they do not both contain any common individual proteins. In (*c*), we assume that only proteins with consecutive indices can form a link in order to form a complex. The left most panel indicates the isolated proteins. The next panels show the possible complexes that can form after one, two or three reactions, respectively. Complexes with one, two, three or four proteins are represented as circles of increasing sizes (coloured blue, orange, green and red, respectively, in the online version). (Online version in colour.)

These three models are depicted in figure 2 for the case *N* = 4, which we will use as an illustrative example because our motivating problem in *Drosophila* contains four proteins. Beginning with the four unbound proteins, figure 2 shows the sequence of reactions needed in order to make all possible complexes allowed under each binding model. Models 1 and 2 lead to the formation of the same intermediate complexes before a full complex containing all four subunits is formed, but they are formed through different reactions. For example, in Model 2, heterodimer 12 may bind with 34 to form the full complex 1234. Such a reaction is not permissible under Model 1. With Model 3, some complexes cannot form, for example the heterodimers 13, 14 and 24 are never produced. Table 1 summarizes all of the complexes that may be formed for the three models.

**Table 1.** Complete list of all protein complexes formed for the case *N* = 4 in the three models. Models 1 and 2 lead to the formation of the same complexes, but not all of these complexes can be formed in Model 3. (Online version in colour.)

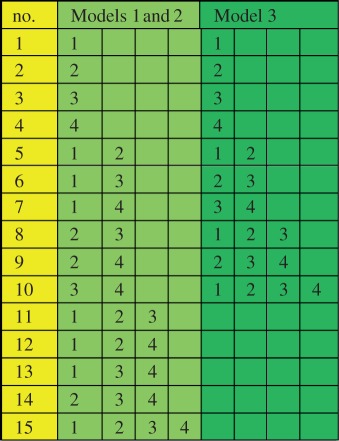

**Figure 3.**
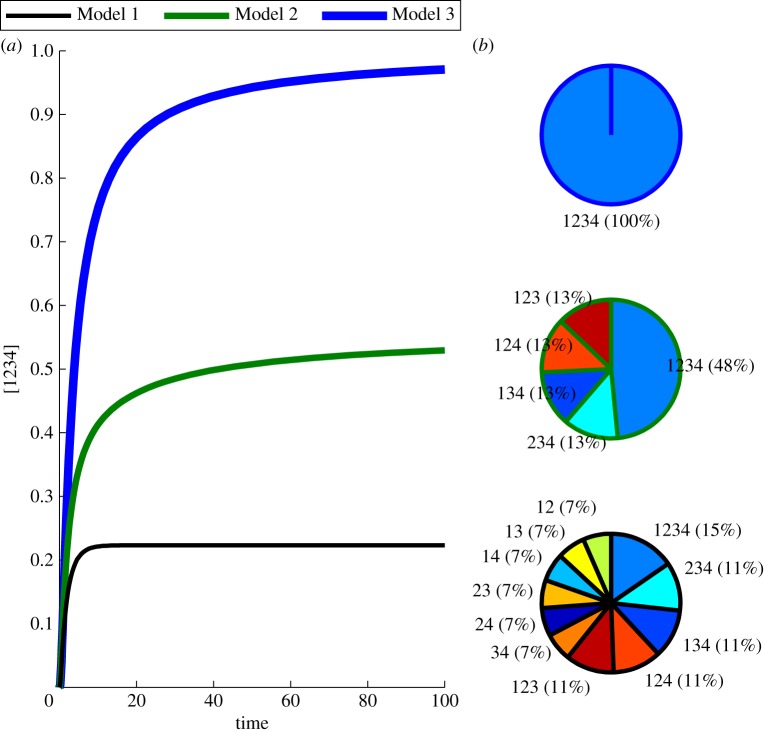
Mechanism of complex formation determines dynamics of tetramer production and prevalence of intermediates. Panel (*a*) shows the monotonic approach of the concentration of the tetramer to its steady-state value for each of the three models. The steady-state composition is shown in panel (*b*) for each of the three models (Models 3, 2 and 1 from top to bottom). With Model 3, the concentration of the tetrameter makes a slow approach to a value near 1, while only small amounts of the intermediate complexes remain. Model 2 results in more intermediate complexes, with some of each of the trimers remaining, while Model 1 results in many dimers and trimers.

For each model, we consider initial concentrations of 1 for each of the four proteins and set all rate constants to 1. All reactions are assumed to occur by standard mass action kinetics. We initially consider only complex formation reactions and do not model transcription, translation or degradation of any of the protein monomers. Thus, no new monomers will be formed, and monomers are lost only to complex formation. Figure 3*a* shows the accumulation of the tetramer 1234 over time under each of the three models. Only in Model 3 does the concentration of the tetramer approach a value near 1, indicating that nearly all the individual proteins eventually participate in the formation of this complex. In the other models, most of the individual proteins are locked up in intermediate complexes that are unable to bind together. This is depicted in figure 3*b*, which shows the steady-state distribution of the protein complexes formed under each of the three models. Models 1 and 2 result in many intermediate complexes that are unable to bind to one another. Under Model 3, on the other hand, almost all monomers are bound in a tetramer.

This simple example illustrates that the mechanism of protein binding strongly effects the distribution and dynamics of complex formation. To apply this principle to study biological rhythms generated by a transcription–translation feedback loop (TTFL), we consider the case that the full tetramer (1234) negatively feeds back on the production of the four monomers, which are also linearly degraded. This leads to differential equations of the form

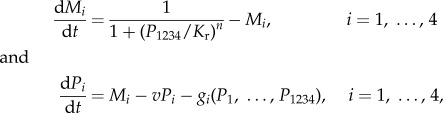
where *M_i_* and *P_i_* are the concentrations of mRNA and protein for monomer *i*, and *v* is the protein degradation rate. The functions *g_i_* describe the mass action binding reactions between *P_i_* and the other species, and differ for the three models of complex formation described previously. We assume that degradation and translation of the transcripts for each protein occur with rate 1. Transcription occurs according to a standard Hill function with Hill coefficient *n* and repression constant *K*_r_. As before, complexes are gained and lost through binding events, but now in addition, they undergo linear degradation with rate constant *v*. We assume saturating degradation for the final tetramer, leading to a differential equation of the form

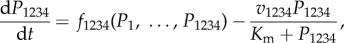
where *f*_1234_ describes the binding reactions which form *P*_1234_, and *v*_1234_ and *K*_m_ are the maximal degradation rate and associated Michaelis–Menten constant, respectively. Our motivation for this is to create a stable final complex, at least for higher concentrations, which is in agreement with the available biochemical data [33].

**Figure 4.**
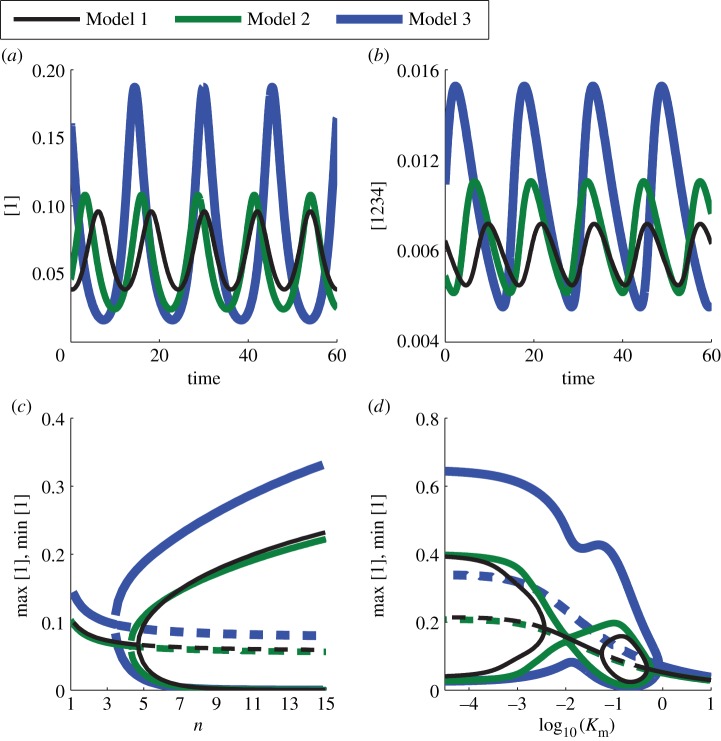
Different oscillatory regions in parameter space are observed under the three models of complex formation when placed in a TTFL. Sample trajectories for (*a*) the first monomer, 1, and (*b*) the final tetrameric complex, 1234, are shown for all three models (*n* = 5, *v* = 0.4, *K*_r_ = 0.004, *K*_m_ = 0.4, *v*_1234_ = 0.05). (*c*) A bifurcation diagram in *n* showing that with a sufficiently high Hill coefficient, oscillations occur in each of the models. (*d*) A bifurcation diagram in *K*_m_ showing that the pattern of onset and offset of oscillations as *K*_m_ is reduced varies between the three models. In the bifurcation diagrams, computed using XPPAUT v. 7.0 [34], a solid line denotes a stable equilibrium or limit cycle and a dashed line represents an unstable equilibrium.

After searching the parameter space, we were able to obtain oscillations in each of the models. We plot the concentration of protein 1 for all three models in figure 4*a* and the concentration of the final tetrameric complex in figure 4*b* for a nominal choice of parameters for which all three models oscillate. The value of the Hill coefficient determines whether oscillations are seen, and their frequency and amplitude. To study this, we show a bifurcation diagram in *n* in figure 4*c*. Oscillations occur as the Hill coefficient is increased. The value of *n* for which oscillations first occur is smallest for Model 3, then Model 2 and finally Model 1. This nesting of the oscillatory region also occurs for the *K*_m_ parameter. In figure 4*d*, we show a bifurcation diagram in *K*_m_, which controls the location of the onset of saturating degradation of the tetramer. All three models oscillate for small *K*_m_ and are non-oscillatory for large *K*_m_. For intermediate *K*_m_ (so that the mean value of the tetramer is near *K*_m_), Models 1, 2 and 3 do not oscillate, oscillate with small amplitude and strongly oscillate, respectively. The nesting of the oscillatory regions for the three models holds true for other choices of nominal parameters as can in part be seen from the 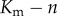 two-parameter bifurcation diagram in the electronic supplementary material, figure S1.

Together, these results show that the mechanism of repressor complex formation in a negative feedback loop strongly affects whether or not rhythms are generated. Moreover, we find that Model 3 (which seems the most likely candidate used in the *Drosophila* circadian clock) has the most likely chance of yielding oscillations.

## 4. Interactions between cells in a network

We next study how interactions between cells within the SCN, the central mammalian circadian pacemaker, can determine the behaviour of the tissue. Previous studies of the circadian timekeeping system have shown that coupling between cells can help heterogeneous neurons with weak, damped oscillations [35,36] or only stochastic oscillations [37] to generate rhythms as a synchronized network. Coupling has also been shown to affect the ability of the network to entrain to external stimuli [38]. Indeed, several previous models of the SCN have been built [2,16,35,36,39–46], but generally many of the details of transcriptional and post-translational regulation are left out. Additionally, the role of coupling between cells in the model is important for fitting parameters, which is often not considered. Models of individual cells are routinely fitted to data from populations. Here, we develop a detailed biochemical model of the SCN, fit parameters that incorporate intercellular coupling to population data, and then compare the result to the same model when coupling is removed. Our goal is to use the model to understand how intercellular signalling leads to emergent phenomena at the network level.

We aim to match experimental measurements of the SCN from both the dispersed cell culture (uncoupled neurons) and organotypic slice preparations (coupled neurons). SCN slices contain a two-dimensional array of neurons, each of which could generate timekeeping. To represent the intracellular rhythms, we use the Kim & Forger model [16] for the mammalian circadian clock, which includes detailed descriptions of complex formation, and transcriptional regulation by the repressor complexes formed. We chose the Kim and Forger model because of its detail and predictive accuracy.

### 4.1. Suprachiasmatic nuclei model formulation

We first extend the Kim and Forger model of intracellular rhythms in the SCN to a tissue-level SCN model by incorporating intercellular signalling. In particular, we focus on the effects of the neuropeptide vasoactive intestinal peptide (VIP), which has been reported to be the most essential signalling molecule in the SCN [20,47–51]. VIP is released by roughly 20% of the neurons of the SCN and received by the cell-surface VPAC_2_ receptor (VPAC_2_R). This receptor has been shown to be present on the surface of upwards of 90%, if not all cells in the SCN [52], so we will assume all cells can receive VIP. Reports about whether VIP is produced rhythmically or not conflict [53–55], but it is generally agreed upon that VIP release should be rhythmic [56], so we focus only on VIP release.

**Figure 5.**
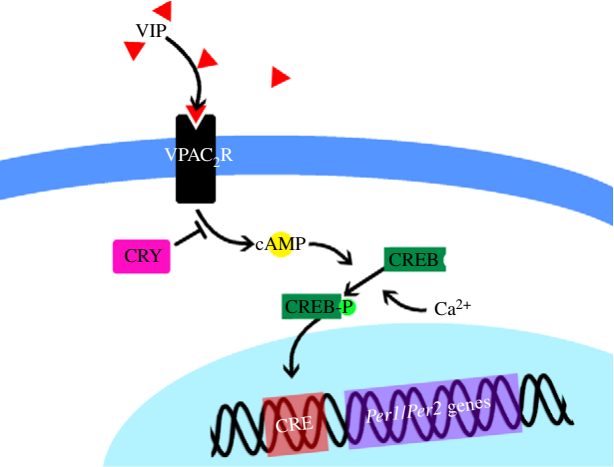
Diagram of the steps of the CRE activation pathway included in the model. Binding of VIP to the cell-surface receptor VPAC_2_R leads to increases in cAMP, which together with intracellular calcium promote the phosphorylation and activation of CREB. CRY non-competitively binds the intracellular domain of VPAC_2_R, inhibiting it. Activated CREB binds to CRE sites on the promoters of the *Per1* and *Per2* genes increasing their transcription.

Binding of VIP to the receptor initiates an intracellular signalling cascade with several steps occurring on a very fast time scale (figure 5). Condensing this fast time scale, we model only the ligand–receptor binding and a few key steps in the pathway for which we have experimental data to compare. Specifically, we assume that VIP binding to the receptor leads to an increase in cAMP, which promotes phosphorylation and activation of cyclic AMP response element binding protein (CREB). The activated CREB then binds to the cyclic AMP response element (CRE) sites in the promoter regions of the *Per1* and *Per2* genes, increasing their transcription [57]. The rate of VIP release is assumed to be proportional to intracellular calcium levels, and calcium can also, through a parallel pathway, promote the phosphorylation of CREB directly [58]. Intracellular calcium is assumed to increase at a rate proportional to E-box activity and decrease at a linear rate. While this is a purely phenomenological assumption, it is consistent with experimentally measured time courses of intracellular calcium, which show the greatest increase roughly at the time of maximal E-box activity [59,60], and creates a phase difference between calcium and the molecular clock components consistent with those found experimentally as well as in previous modelling studies [43]. Finally, CRY1 and CRY2 are assumed to inhibit the adenylyl cyclase activity of VPAC_2_R [61]. This pathway is diagrammed in figure 5, and all equations, variables, and parameters modified from the original Kim and Forger model, or added to make this new model are given in the electronic supplementary material, S2.

### 4.2. Parameter estimation and simulating the suprachiasmatic nuclei model with graphics processing units

**Figure 6.**
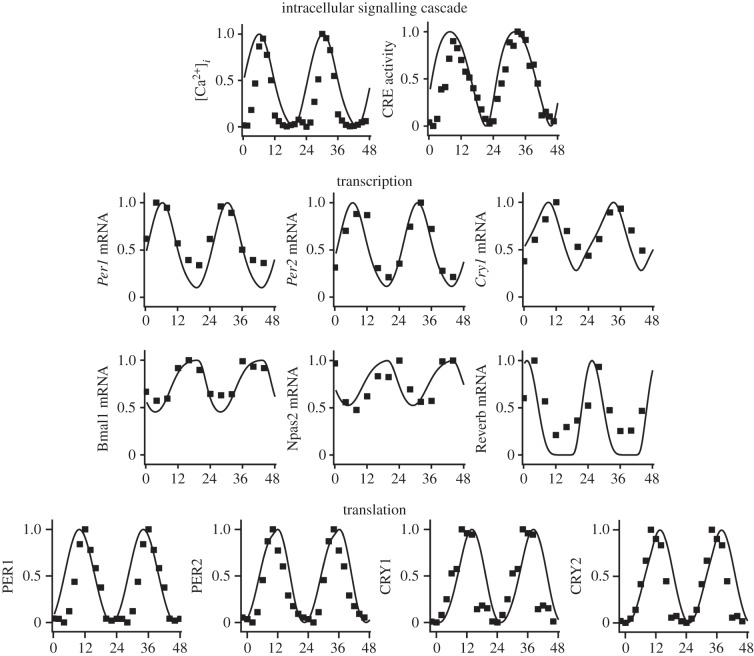
Fits of the extended single-cell model to mRNA, protein, CRE activity and Ca^2+^ time series. Model predictions are shown as smooth curves, and experimental data as squares. All time series are normalized by their maximum values. mRNA and protein time series are the same as used in [16]. CRE and Ca^2+^ time series extracted from videos in [59].

The addition of intercellular signalling to the Kim and Forger model requires the fitting of 15 new parameters. To represent a single cell, *Per1* and *Per2* maximal E-box transcription rates (parameters trPo and trPt) were set to 40% of their originally published maximal values, as recommended in the original manuscript, and all other original parameters were unchanged [16]. This was done to allow for additional *Per* transcription initiated by CRE activation through VIP and calcium. The 15 new parameters were fitted using simulated annealing as in the original Kim and Forger publication [16]. In addition to the protein and mRNA time courses and protein abundance data that the original model was fitted to, we additionally fit to time courses of intracellular calcium and CRE activity levels, extracted from published videos [59] using Matlab. To fit parameters for a single cell in the SCN network, we coupled the cell to itself so that it received the VIP that it produced. This allowed us to fit the activation of the CRE pathway by VIP in a single cell. For simulation of the whole SCN, the VIP released by each cell was divided by the number of VIP-producing cells, so that the input signal from the network was of the order of the originally fitted VIP level. Further details on the methods of parameter estimation are given in the electronic supplementary material, S3.1. Fits of the model to experimental data obtained are shown in figure 6.

We simulate an SCN network model of 1024 cells in total and assume that VIP is produced by 205 of the cells (approx. 20%). VIP is a small neuropeptide, which should diffuse rapidly throughout the SCN compared with the time scale of transcriptional activation. Therefore, we make the simplifying assumption that the VIP-producing cells released VIP equally to all cells in the SCN. To test the consequences of this assumption, we compared the time courses obtained with this connectivity to those seen with random network connectivity of varying percentages ranging from 10 to 100%, normalizing the VIP input to each cell by the number of upstream cells, and saw no significant difference in synchrony, period, amplitude or waveform (data not shown).

Heterogeneity is included in the model by allowing the *Per1* and *Per2* maximal transcription rates associated with both E-box activation (parameters trPo and trPt, respectively) and CRE activation (parameters CtrPo and CtrPt, respectively) to vary between cells. For each cell, these four parameters were drawn independently from normal distributions with mean equal to the value from our fitted single-cell model, and standard deviation 5% of the mean. This affects the strength of oscillations seen in individual cells as well as their intrinsic periods of oscillation. Only these transcription rates were varied between cells; all other parameters were set to the values fitted as described above.

Both the parameter estimation algorithm and simulation of the network model are accelerated using GPU computing. This architecture is well suited for these problems because of their structure. Parameter estimation using simulated annealing is embarrassingly parallel and fits entirely within the memory onboard the GPU card. Simulation of the SCN network is also sufficiently small to fit in memory; however it does require some communication between threads. In particular, information about intercellular coupling through VIP must be passed between cells, but this is a small amount of data relative to the computation of the intracellular dynamics, which can be solved in independent blocks. For our problem, we choose to simulate 1024 cells in parallel, either uncoupled for parameter estimation, or coupled for the SCN network model, because tests showed that this was a near optimal choice in terms of speed of computation per number of cells (see the electronic supplementary material, figure S2). Details of the computational methods used are given in the electronic supplementary material, S3.

### 4.3. Differences between single cell and whole suprachiasmatic nuclei behaviour

**Figure 7.**
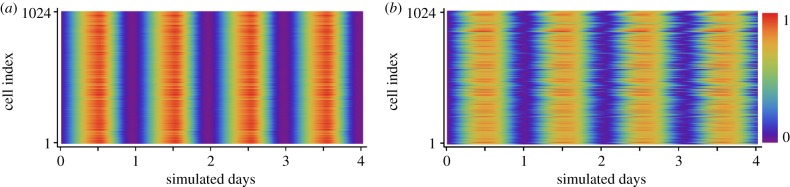
PER2 rhythms in the simulated SCN. (*a*) Raster plots of 200 cells selected randomly from the 1024 cells in the simulated SCN. Each row of the raster plot shows the total PER2 protein level versus time for one cell. Cells are all well synchronized throughout the simulation. (*b*) Raster plots from a simulation of uncoupled cells show that without VIP signalling, neurons drift out of phase as time progresses due to differences in intrinsic period. PER2 levels are normalized within each panel and displayed according to the colour scheme at the right. Cells are indexed from top to bottom in random order.

We consider our model both in the presence and absence of coupling through VIP. Raster plots of PER2 levels for 200 randomly selected cells (out of 1024) from the coupled SCN model are shown in figure 7*a*. Cells within the intact SCN have well-synchronized, high-amplitude rhythms. The uncoupled model mimics the dispersed cell preparations often used experimentally. In these simulations, the VIP release rate (parameter vpr) is set to zero, as cells are assumed to be dispersed enough that they are unable to signal through VIP release. Raster plots of PER2 levels for the uncoupled model are shown in figure 7*b*. In the absence of coupling, the cells desynchronize and show a drastic reduction in rhythm amplitude, with some losing rhythmicity altogether. While the uncoupled cells all begin with similar initial conditions, they drift apart over time due to their differences in intrinsic periods. Periods of individual cells range from 24.0 to 25.6 with a mean of 24.79 ± 0.29 and are distributed roughly normally. Of the 1024 isolated cells simulated, 966 showed sustained rhythms while the other 58 were arrhythmic or heavily damped.

Consistent with the original model [16], we find that our uncoupled model matches known single-cell genotypic knockouts: 70% of *Per1*^−/−^ and all *Cry1*^−/−^ cells modelled were arrhythmic, while *Cry2*^−/−^ cells were mostly rhythmic (918 of 966 rhythmic) with a long period (mean 27.13 ± 0.38 h). This is all consistent with experimental data for SCN neurons in dispersed culture [23]. In the coupled SCN model with intact VIP signalling, cells synchronize with a period of 24.2 h, consistent with SCN explants [23]. Unlike the isolated cells, the network model is resistant to genotypic knockouts, showing robust rhythms in *Per1*^−/−^, *Cry1*^−/−^ and *Cry2*^−/−^ individual knockouts, with no period change, period shortening by approx. 1 h, and period lengthening by approx. 1.5 h, respectively, for the three knockouts.

**Figure 8.**
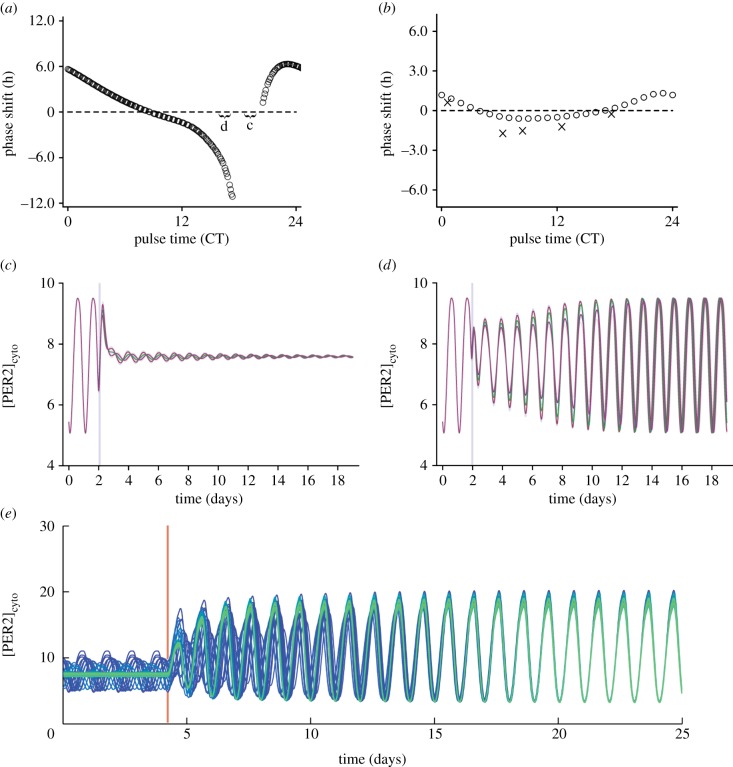
Effect of VIP on individual cells and the SCN network. PRCs predicted by the model for (*a*) a single cell and (*b*) the SCN network (circles) in response to a VIP pulse show that single cells can be shifted much more easily than the SCN network (note the difference in scales). Experimentally determined phase shifts for application of 100 nM VIP to the SCN from [47] are labelled as cross symbols in (*b*) for comparison. VIP pulses given to the single cell (labelled as regions c and d in (*a*) and denoted by grey vertical lines) can either (*c*) cause the cell to become arrhythmic or (*d*) phase shift it. In particular, pulses between CT17.3 and CT20.5 cause the cell to lose rhythms, shown as a gap on the PRC (single-cell parameters are given in the electronic supplementary material). (*e*) Restoring VIP signalling (denoted by a red vertical line) to an uncoupled network of half rhythmic (blue), half arrhythmic (green) cells quickly restores rhythms and synchrony to the network.

To test the robustness of individual cellular rhythms, we constructed a phase response curve (PRC) to VIP to see how they phase change in response to external signalling. For this curve, circadian time was determined relative to the cell's PER2 protein level, with the peak PER2 level defined as CT12. Individual cells can show both large phase advances as well as delays depending on the time at which the VIP is applied, and surprisingly can also be made arrhythmic (denoted as a gap in the PRC). A PRC for a single cell is shown in figure 8*a*. For this particular cell, a VIP pulse between CT17.3 and CT20.5 causes the cell's rhythms to damp (figure 8*c*), in stark contrast to the quickly recovered rhythms when the pulse is given at other circadian times (figure 8*d*). This bistability was surprising, as it had not been seen with the parameters used in the original Kim and Forger model, and is a novel prediction of our new single-cell model. It is, however, consistent with experiments which have shown that application of forskolin, a direct stimulant of the cAMP signalling pathway, to isolated SCN neurons can cause the cells to gain or lose rhythmicity [22]. Other models have also found this behaviour, notably in the light response of the *Drosophila* circadian clock [29] and in a particular range of parameters in an overall model of mammalian circadian rhythms [62].

In our model, bistability is a property of the individual cellular oscillators, but not of the SCN network. VIP pulses of identical magnitudes applied to the SCN network show much smaller phase shifts, with a traditional type I PRC, as described in [32], shown in figure 8*b* (note the difference in scale). Thus, the intercellular coupling confers robustness against external perturbations. The SCN network PRC qualitatively matches that found experimentally [47], with roughly the same pattern of advances and delays. The main notable difference is that the magnitude of the phase delays is not as great as in [47], which could be explained by the pharmacokinetics of VIP, or small differences in the magnitude of the VIP pulse given, as compared to our simulations.

To explore this bistability in single cells further, we tested whether the reintroduction of VIP signalling could restart collective rhythms in the network. Using our uncoupled model from before, we began with half the cells in the rhythmic state and the other half in the arrhythmic state, and reintroduced VIP signalling. Plots of 32 representative cells from the 1024 in the network with varying initial amplitudes (with bright to dark colours representing low to high initial amplitudes, respectively) and phases are shown in figure 8*e*. After restoring VIP signalling (time denoted by the red vertical line), all of the cells in the network quickly regained high-amplitude rhythms and resynchronized over time. This illustrates the fact that bistability is not seen in the coupled network, but is seen in isolated cells.

## 5. Discussion

Here, we have shown that the details of how parts of a rhythm-generating system interact play a crucial role in determining the behaviour of biological clocks. These details are often not included in the summary diagrams biologists use to describe biochemical interactions. Our results demonstrate that they are crucially important at every step of rhythm generation in a genetic network; from transcriptional regulation, to post-translational modification, and finally to intercellular communication. We also show that much more investigation may be needed to determine the parts of timekeeping networks that are crucial for behaviour.

Combinatorial complexity is seen in many biological systems. This is because almost all cellular processes involve protein complex formation. It is thus surprising that so few studies have included the details of protein complex formation. We find that they can be crucial in correctly predicting a system’s behaviour. Many of the behaviours we have described such as chaos and bistability have been seen in more complex models of biological timekeeping [63–65]. By studying a simple negative feedback loop, we show how they can be attributed to the way in which transcriptional regulators interact, an often overlooked but essential step in rhythm generation.

The development of detailed multicellular models of biological tissues has the potential to explore many complicated phenomena seen experimentally, and to try to explain the mechanisms behind them. An important area for future work is to study the differences in phase among neurons in the SCN network. Heterogeneity of period and phase within the SCN has been shown to be important for the coding of seasonal changes in light and dark cycles [66–68]. Previous studies have shown that cells in subregions of the SCN exhibit differences in intrinsic period [66,69–71], and some have suggested that these period differences drive the spatio-temporal pattern or ‘phase wave’ seen in SCN slices [71].

With our SCN model, we focused on the molecular mechanisms of rhythm generation. In the actual SCN, there is a strong connection between the molecular clock and the electrical activity of the cells [72], and previous studies have shown that the rhythms in electrophysiology can strengthen molecular rhythms [43]. While we have not considered this aspect of rhythm generation here, we plan to explore it in future work.

Here, we show how coupling can make the SCN more resistant to perturbations, which is in agreement with experimental findings [73]. Our findings also show how coupling greatly increases the amplitude of rhythms, which also matches experimental findings [20,74]. But most interestingly, we find that when cellular coupling is included in model fitting, individual cells lacking the coupling signal become bistable. This matches data which show that timekeeping within individual isolated neurons within the SCN can be turned on or off by biochemical signals. It also raises questions about our single-cell simulations of circadian mutants and the data that we compare our simulations against [23]. When single cells are arrhythmic, it remains to be seen if that is due to bistability or if the cells are incapable of rhythmicity. This needs to be explored both in simulations and in the experimental preparation.

The designs we have tested are inspired by the experimental findings from the circadian clock found in several organisms. While more work is needed, our findings point towards circadian clocks using mechanisms that yield robust oscillations. The results presented here are numerical, and further mathematical analysis could expand these results [75]. The core negative feedback loop that generates timekeeping in many organisms is controlled by one or multiple E-boxes, which are repressed by elements in the feedback loop. We find this design can yield bistability, but not chaos. Additionally, we find that bistability in intracellular timekeeping, which makes timekeeping vulnerable to perturbations, can be compensated for by intercellular coupling.

Further experimental work is needed to verify our predictions. It would be interesting to build a synthetic clock where a protein, under negative feedback, acts both as an activator and repressor. Such a design could show behaviours not yet seen in synthetic clocks, especially as the araC protein, which can show this behaviour, has already been included in a synthetic clock. We also predict that individual isolated SCN neurons are bistable. This matches data presented in Webb *et al.* [22], but better experimental data would characterize the single-cell PRC to VIP and the possibility that it could stop rhythmicity, offering experimental validation of our prediction. We also note that the protein binding scheme that is most likely to show oscillations matches what is currently known about NMO, PER, TIM and SGG, at least that NMO binds PER, PER binds TIM and TIM binds SGG [18,19]. However, the interactions between these four proteins are not yet fully worked out, which could be validated by a two-hybrid system followed by a detailed biochemical study. While the details of these interactions could be difficult to determine, our results indicate that their importance makes this effort worthwhile.
